# Supplementary material for: Building molecular model series from heterogeneous CryoEM structures using Gaussian mixture models and deep neural networks
Source: Commun Biol. 2025 May 25;8:798. doi: 10.1038/s42003-025-08202-9 (PMC12104439; doi:10.1038/s42003-025-08202-9)
Supplement: Supplementary file 1 — Supplementary Information [file 42003_2025_8202_MOESM1_ESM.pdf]

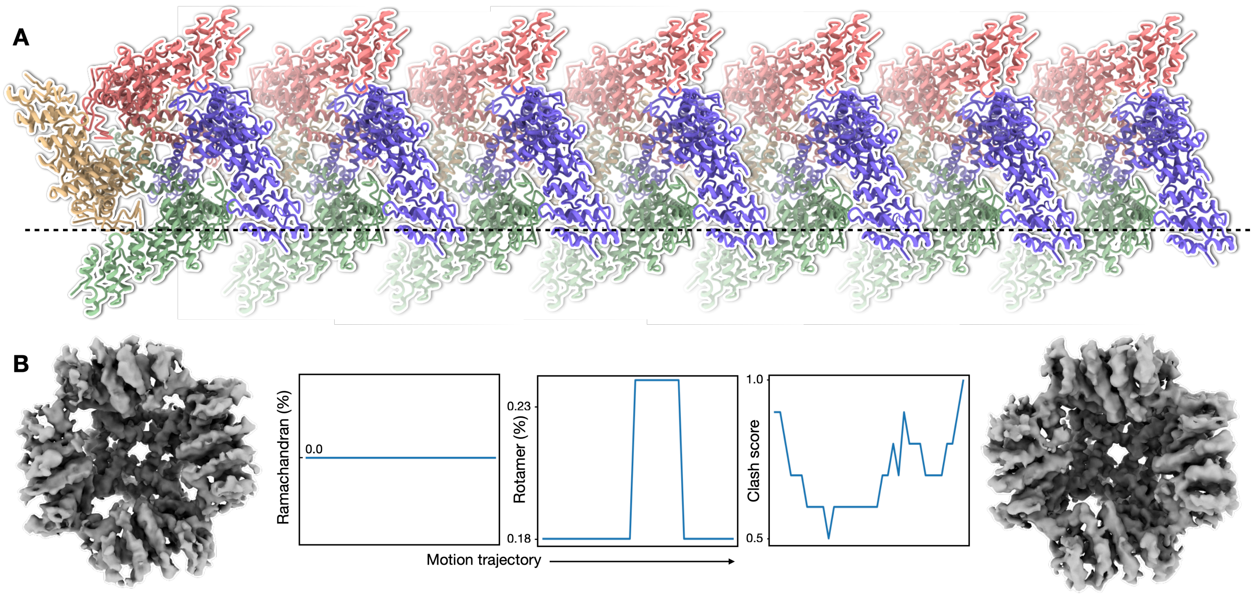

Figure S1. Model series refinement of TRPV1. **(A)** Snapshots of GMM refined molecular model series along one continuous motion trajectory of TRPV1 from dataset EMPIAR-10059. **(B)** Left and right panels - 3D reconstruction of the first and last frame of the motion; middle three panels - Ramachandran, rotamer outlier, and clash score for models along the motion trajectory.

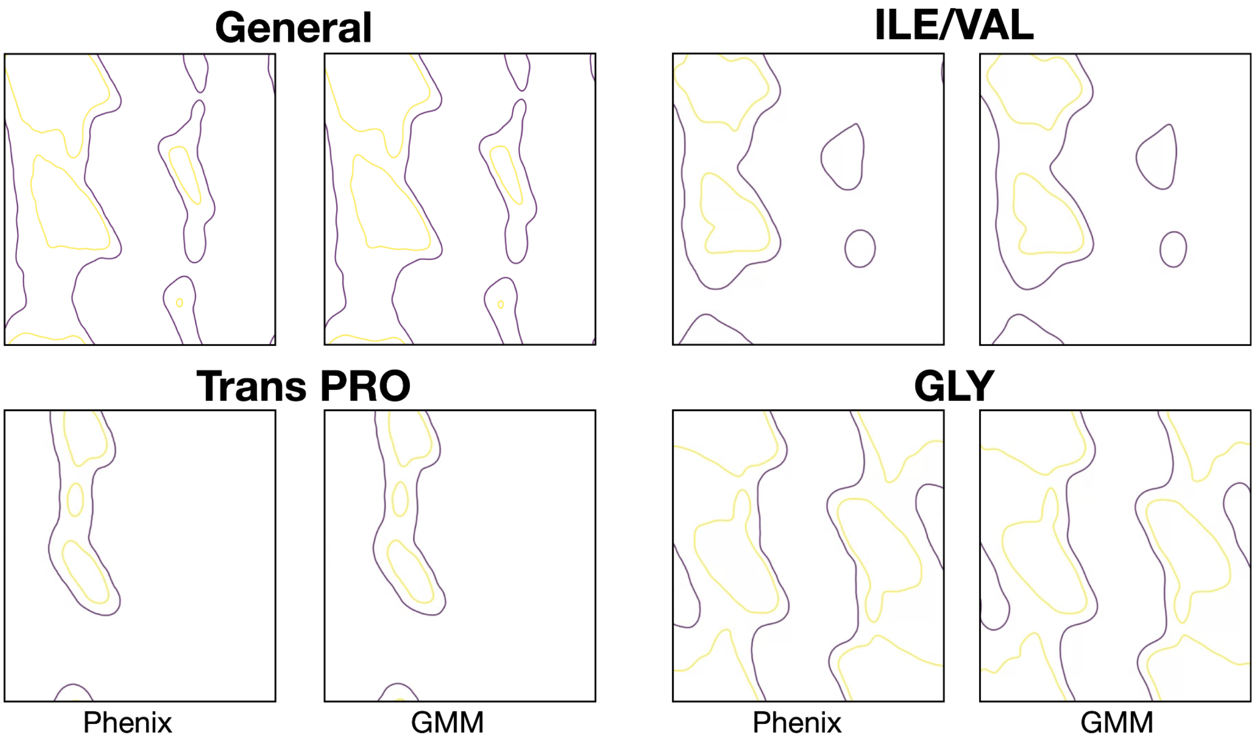

Figure S2. Comparison between histogram representation of the Ramachandran plot from Phenix, and the GMM representation of the corresponding plot. Four types of Ramachandran plots are shown, and the contour lines indicate the boundary of allowed (yellow, 2%) and outlier (purple, 0.05%). The Phenix and GMM plots of the same type should be nearly identical.

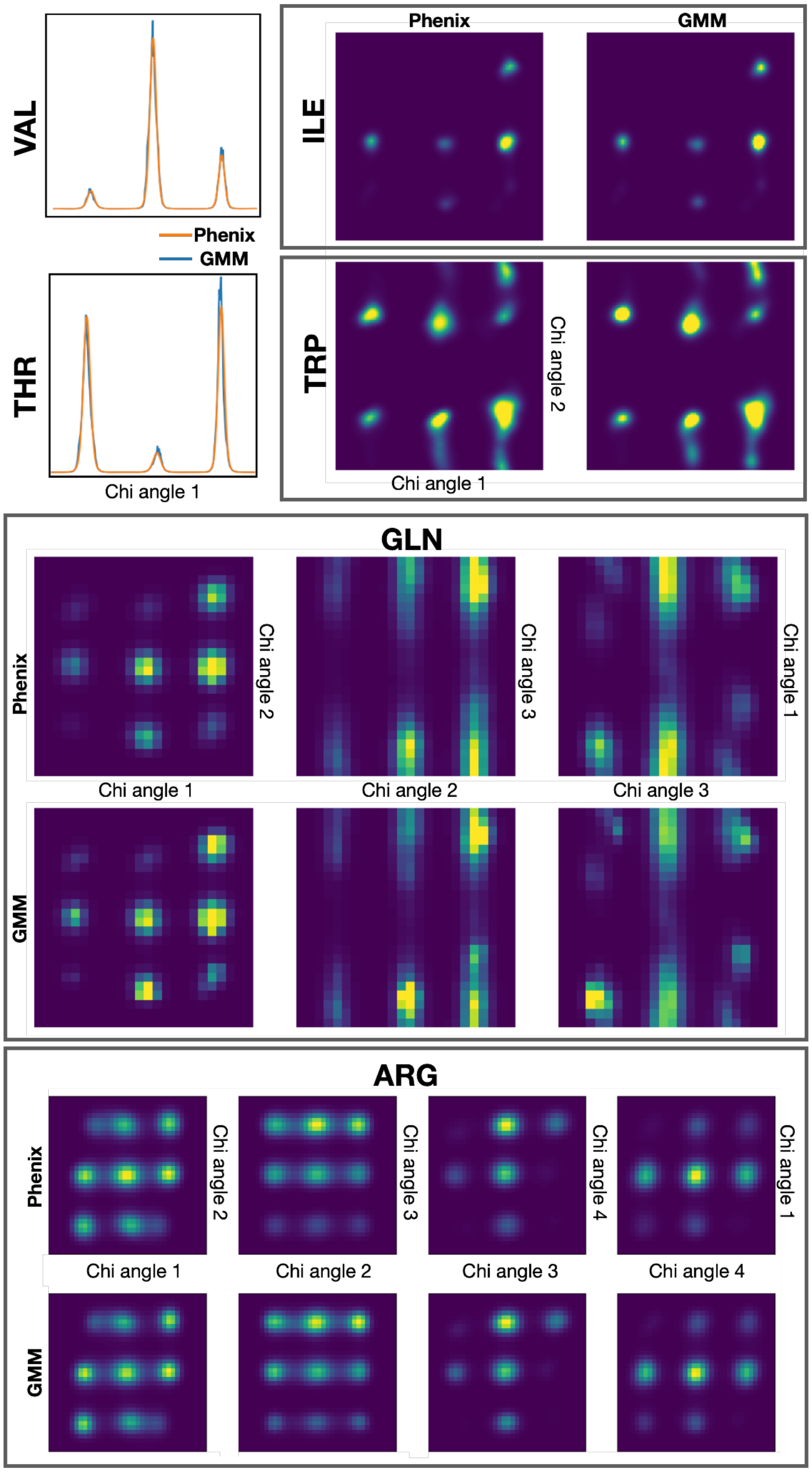

Figure S3. Comparison between histogram representation of the preferred rotamer from Phenix, and the GMM representation of the corresponding plot. Here we show two types of sidechains with one Chi angle (VAL, THR), two types with two Chi angles (ILE, TRP), one type with three Chi angles (GLN), and one with four Chi angles (ARG).

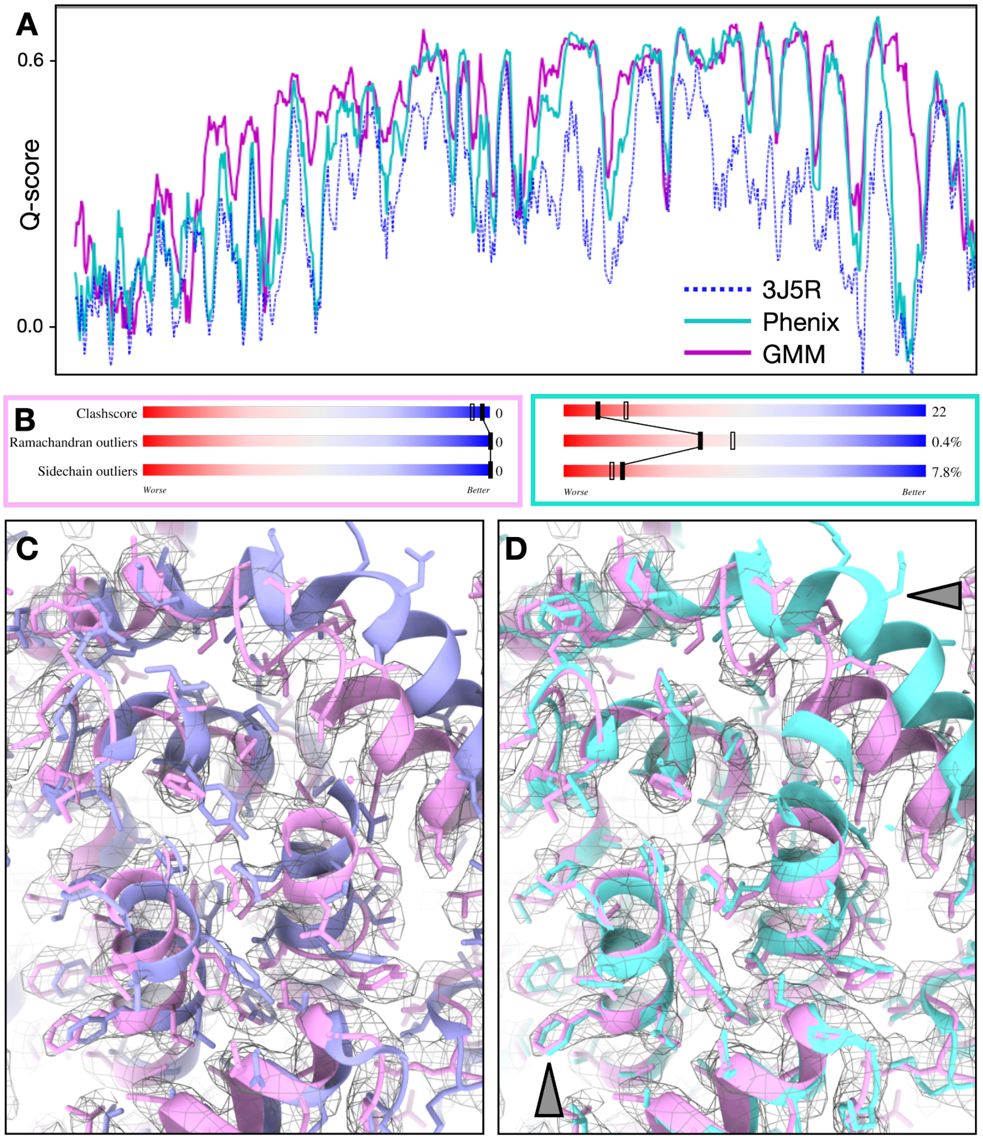

Figure S4. Comparison between the GMM-based model refinement and Phenix real-space refinement in the TRPV1 example shown in Figure 1. **(A)** Q-score comparison. Blue - original PDB model; cyan - Phenix real space refinement; pink - GMM-based refinement. **(B)** PDB validation metric comparison. Left - GMM-based refinement; right - Phenix real-space refinement. **(C)** Overlay of the original (blue) and GMM refined (pink) model with the CryoEM map. **(D)** Overlay of the Phenix refined (cyan) and GMM refined (pink) model with the CryoEM map. The arrow points to a helix that was not fit into the map by the Phenix real-space refinement.

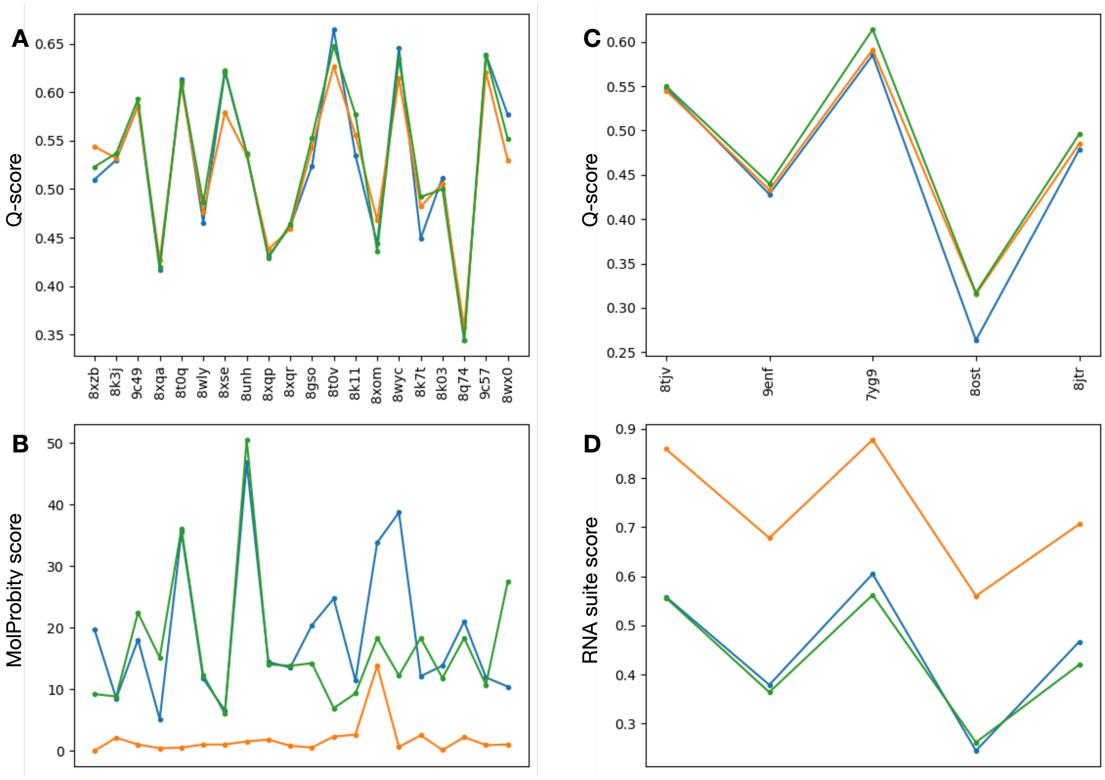

Figure S5. Comparison between the GMM-based model refinement and Phenix real-space refinement on protein and RNA examples. Blue - original PDB model; green - Phenix real space refinement; orange - GMM-based refinement. **(A)** Q-score comparison for 20 protein structures. **(B)** MolProbity score comparison for 20 protein structures. Note lower MolProbity score is better. **(C)** Q-score comparison for 5 RNA structures. **(D)** RNA suite score comparison for 5 RNA structures.

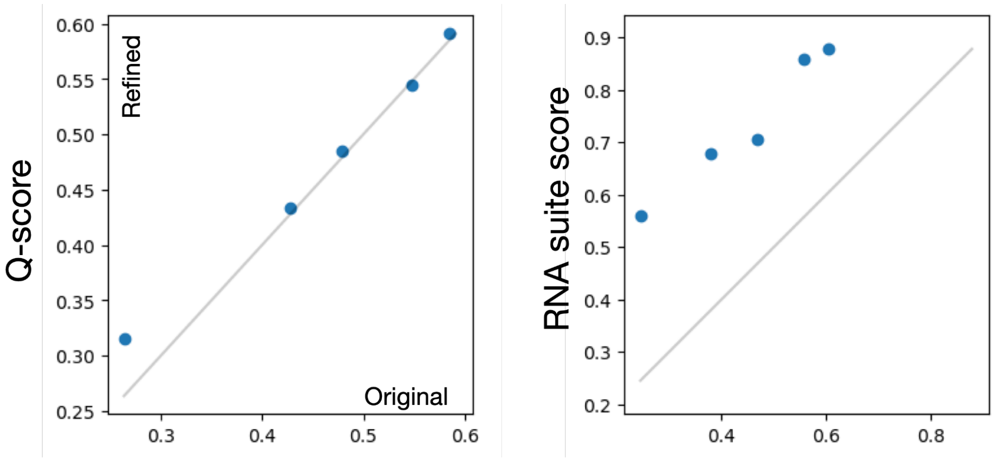

Figure S6. Comparison of the original and refined version of 5 models with RNA from the PDB. Left - Q-score; right - RNA suite score (higher score indicates better RNA backbone geometry).

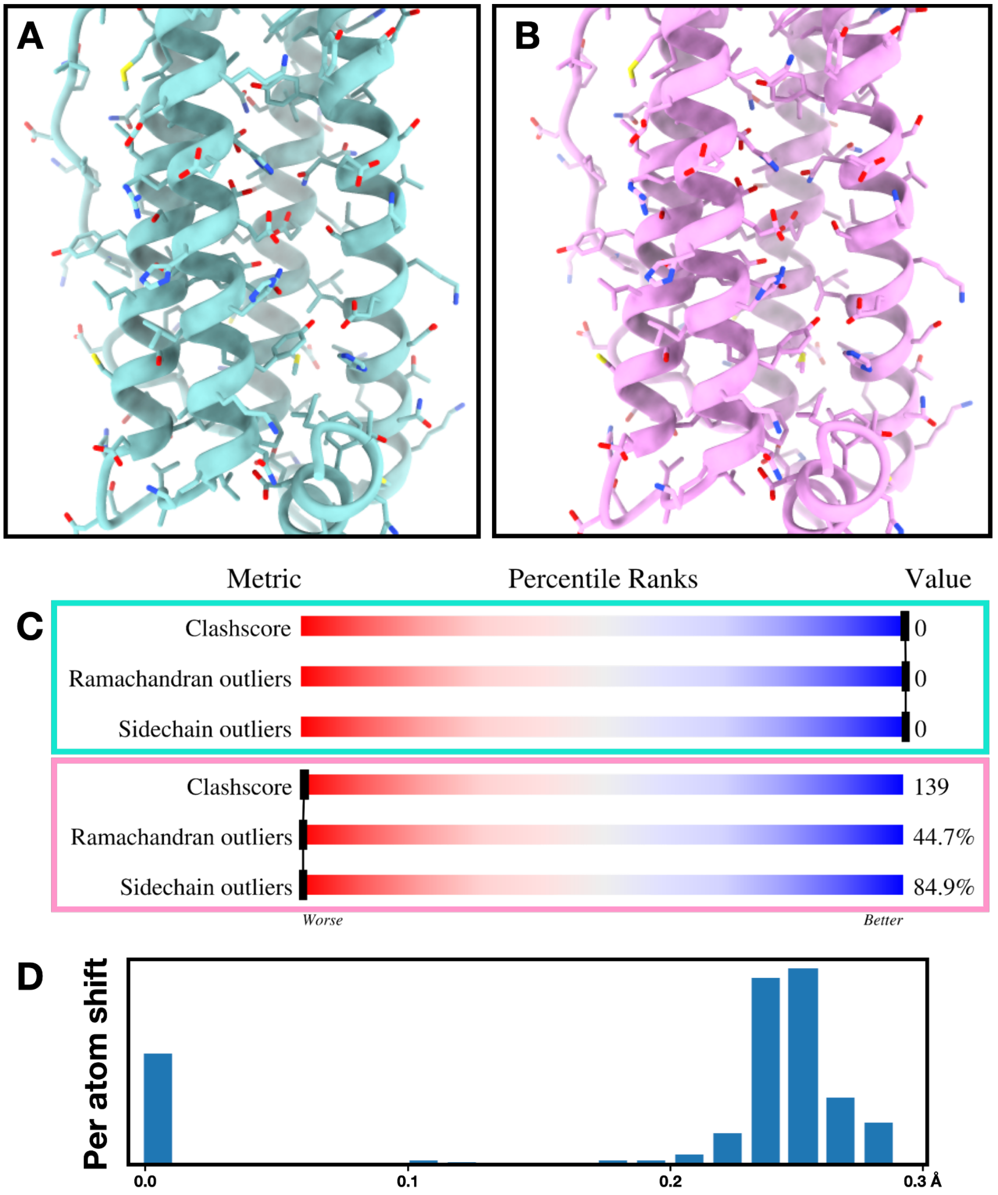

Figure S7. Sensitivity of the PDB validation metrics. (A) Input model of apoferritin (PDB-8T4Q). (B) Output model from the GMM-based refinement with an inverse loss function, using the model from A as input. (C) Comparison of PDB validation scores between the input (top) and inverse "refined" model (bottom). (D) Histogram of per-atom shift distance between the two models.
