# Supplementary material for: Building molecular model series from heterogeneous CryoEM structures using Gaussian mixture models and deep neural networks
Source: Commun Biol. 2025 May 25;8:798. doi: 10.1038/s42003-025-08202-9 (PMC12104439; doi:10.1038/s42003-025-08202-9)
Supplement: Supplementary file 2 — Description of Additional Supplementary Files [file 42003_2025_8202_MOESM2_ESM.pdf]

Description of Additional Supplementary Files

File name: Supplementary video 1

Description: Model series generated from the TRPV1 dataset (EMPIAR-10059), along a linear trajectory in the conformational space.

File name: Supplementary video 2

Description: Model series generated from the spliceosome dataset (EMPIAR-10180), along a linear trajectory in the conformational space.

File name: Supplementary video 3

Description: Model series generated from the spliceosome dataset (EMPIAR-10180), along a circular trajectory in the conformational space.
